# Coherent Spin Dynamics of Electrons in CsPbBr_3_ Perovskite Nanocrystals at Room Temperature

**DOI:** 10.3390/nano13172454

**Published:** 2023-08-30

**Authors:** Sergey R. Meliakov, Evgeny A. Zhukov, Evgeniya V. Kulebyakina, Vasilii V. Belykh, Dmitri R. Yakovlev

**Affiliations:** 1 P.N. Lebedev Physical Institute of the Russian Academy of Sciences, 119991 Moscow, Russia; 2 Experimentelle Physik 2, Technische Universität Dortmund, 44227 Dortmund, Germany

**Keywords:** perovskite nanocrystals, CsPbBr3, coherent spin dynamics, electron and hole g-factors, time-resolved Faraday rotation

## Abstract

Coherent spin dynamics of charge carriers in CsPbBr${}_{3}$ perovskite nanocrystals are studied in a temperature range of 4–300 K and in magnetic fields of up to 500 mT using time-resolved pump-probe Faraday rotation and differential transmission techniques. We detect electron spin Larmor precession in the entire temperature range. At temperatures below 50 K, hole spin precession is also observed. The temperature dependences of spin-related parameters, such as Landè *g*-factor and spin dephasing time are measured and analyzed. The electron *g*-factor increases with growing temperature, which can not be described by the temperature-induced band gap renormalization. We find that photocharging of the nanocrystals with either electrons or holes depends on the sample cooling regime, namely the cooling rate and illumination conditions. The type of the charge carrier provided by the photocharging can be identified via the carrier spin Larmor precession.

## 1. Introduction

Lead halide perovskite semiconductors are the focus of research interest due to their exceptional photovoltaic efficiency and optoelectronic properties [1,2,3]. The simple fabrication technology makes them attractive for applications as solar cells, light emitting devices, radiation detectors, etc. They also demonstrate remarkable spin properties, facilitating spintronic applications [3,4,5,6]. Colloidal nanocrystals (NCs), which have been successfully synthesized from the lead halide perovskite, greatly increase the possibilities for tailoring of the material properties [7,8]. Among them, the fully-inorganic CsPbBr${}_{3}$ NCs are mostly investigated and appreciated for their stability.

The spin physics of halide perovskite semiconductors is an emerging research field, which exploits experimental techniques and physical concepts developed for spins in conventional semiconductors [9,10]. Most of the optical techniques developed for the investigation of spin properties and spin dependent phenomena work well for perovskite crystals, polycrystalline films, nanocrystals, and two-dimensional materials. These are optical orientation [11,12,13], optical alignment [12], polarized emission in magnetic field [14,15,16], time-resolved Faraday/Kerr rotation [17,18], time-resolved differential transmission [11,19,20], spin-flip Raman scattering [21,22], and optically-detected nuclear magnetic resonance [23,24,25]. The reported spin dynamics cover huge time ranges from a few picoseconds at room temperature [11,19] up to tens of nanoseconds for spin coherence [25] and spin dephasing [23] times and further up to sub-millisecond times for longitudinal spin relaxation times [24] at cryogenic temperatures.

Experiments on coherent spin dynamics by means of time-resolved pump-probe Faraday/Kerr rotation reveal signals from electrons and holes provided by photocharging in CsPbBr${}_{3}$ NCs [26]. The hole spin coherence has been observed up to room temperature [27], and its optical manipulation has been implemented [28]. The electron spin coherence has not been demonstrated so far for perovskite NCs at room temperature. The spin mode-locking effect reported recently for CsPb(Cl,Br)${}_{3}$ NCs in a glass matrix demonstrates that the elaborated protocols of coherent spin synchronization can be implemented in perovskite NCs [25]. Still, the understanding of spin relaxation and spin decoherence mechanisms is far from being complete.

The Landè *g*-factor controlling the Zeeman splitting of charge carriers and excitons is a key parameter in spin physics. We have recently shown that the universal dependences of the electron, hole, and exciton *g*-factors on the band gap energy are predicted in bulk lead halide perovskites [21,29]. Theoretical analysis predicts that, in NCs, additional mixing of the band states provides a considerable contribution to the electron *g*-factor by deviating it from the universal dependence for bulk; however, the mixing has only a weak effect on the hole *g*-factor [30]. We have confirmed this through low-temperature measurements of CsPbI${}_{3}$ NCs in glass. The temperature dependence of the carrier *g*-factors in perovskite NCs has not been studied so far. Based on this simple approach, one expects that *g*-factor is controlled by the temperature shift of the band gap energy. However, it has been shown for GaAs and CdTe semiconductors that temperature dependence of the electron *g*-factor may have other strong contributions [31,32,33,34], which have an origin that is not yet fully clarified, even for conventional semiconductors. This motivates us to examine this problem for perovskite NCs.

In this paper, we study the coherent spin dynamics of carriers in perovskite CsPbBr${}_{3}$ NCs by time-resolved pump-probe Faraday rotation and differential transmission techniques. The spin dynamics are measured in the temperature range of 4–300 K, where the carrier *g*-factors and spin relaxation times are evaluated. We find an unexpected temperature dependence for the electron *g*-factor. Although at room temperature we observe only electron spin precession, at low temperatures, the hole component may appear. We demonstrate that the photocharging of the NCs with either electrons or holes depends on the sample cooling regime, namely cooling rate and illumination conditions.

## 2. Materials and Methods

### 2.1. Samples

For this study, we use solution-grown lead halide perovskite CsPbBr${}_{3}$ NCs with two sizes of 4.60 nm (sample #1) and 4.74 nm (sample #2). The NCs were synthesized using a procedure described in Refs. [35,36]. A total of 1–1.5 mg of the obtained NCs were dissolved in 60 μL of 3.3 wt% polysterene solution in toluene, and 10 μL of the obtained solution were drop-casted on 5×5 mm glass substrate and dried at room temperature overnight.

### 2.2. Photoluminescence and Optical Transmission

For optical experiments the samples were placed in a helium-flow cryostat, where the temperature was varied from T=4 K up to 300 K. Photoluminescence (PL) was excited by a continuous-wave laser with a photon energy of 3.061 eV (wavelength of 405 nm) and a power of 1.2 mW (excitation density of 1 W/cm${}^{2}$). The PL spectra were measured with an 0.5 m spectrometer and a charge-coupled device (CCD) camera. Optical transmission was measured with a halogen lamp. The transmitted signal was normalized to the intensity of the light sent directly to the detector. The PL and transmission spectra were recorded from the sample area with a diameter of approximately 300 μm.

### 2.3. Time-Resolved Faraday Rotation

To study the coherent spin dynamics of carriers, we used the time-resolved pump-probe technique with detection of the Faraday rotation (FR) [37]. This technique was successfully used for investigation of bulk perovskite semiconductors [17,18,21,23,38] and CsPbBr${}_{3}$ NCs [26,27,28]. Spin-oriented electrons and holes were generated by circularly polarized pump pulses. The laser system used (light conversion) generated 1.5 ps pulses with spectral width of about 1 meV at a repetition rate of 25 kHz (repetition period 40 μs). The laser beam was split into pump and probe beams, which had coinciding photon energies. The time delay between the pump and probe pulses was controlled by a mechanical delay line. The pump beam was modulated with an electro-optical modulator between $\sigma^{+}$ and $\sigma^{-}$ circular polarizations at a frequency of 26 kHz. The probe beam was linearly polarized. The Faraday rotation angle of the probe beam, which was proportional to the carrier spin polarization, was measured as a function of the delay between the pump and probe pulses using a balanced photodetector connected to a lock-in amplifier synchronized with the modulator. The pump power density was tuned to the range $P_{\mathrm{pump}}$ = 3.8–12.7 W/cm${}^{2}$ and the probe power density was $P_{\mathrm{probe}}$ = 3.8–10 W/cm${}^{2}$. The probe beam spot size on the sample was slightly smaller than the size of the pump beam spot, which was about 100μm in diameter. For the time-resolved FR measurements, the samples were placed in a helium-flow optical cryostat and the temperature was varied in the range of 4–300 K. A magnetic field up to 500 mT was applied perpendicular to the laser wave vector (Voigt geometry, B⊥k) by means of an electromagnet.

### 2.4. Time-Resolved Differential Transmission

We used a time-resolved differential transmission (DT, ΔT/T) technique to study population and spin dynamics of charge carriers and excitons at room temperature and zero magnetic field. For measuring the population dynamics, the pump and probe beams were linearly polarized. The pump beam intensity was modulated with an electro-optical modulator at a frequency of 26 kHz. The probe beam transmitted through the sample and the reference laser beam were sent to the balanced photodetector connected with the lock-in amplifier synchronized with the modulator.

For measuring the spin dynamics, circularly polarized pump and probe beams were used. This allowed us to measure the dynamics of the optical spin orientation degree ($P_{\mathrm{oo}}$) at a picosecond time resolution. The pump beam had $\sigma^{+}$ circular polarization. The probe beam was polarized either $\sigma^{+}$ or $\sigma^{-}$, and its intensity was modulated with a mechanical chopper at a frequency of 2 kHz synchronized with the lock-in amplifier. The optical orientation degree is calculated for the $\sigma^{+}$ polarized pump with:

(1) $$P_{\mathrm{oo}}\left(t\right)=\frac{I^{+}\left(t\right)-I^{-}\left(t\right)}{I^{+}\left(t\right)+I^{-}\left(t\right)}\;.$$

Here, $I^{+}\left(t\right)$ and $I^{-}\left(t\right)$ are the signal intensities measured at $\sigma^{+}$ and $\sigma^{-}$ polarizations of the probe beam, respectively.

## 3. Results and Discussion

### 3.1. Photoluminescence and Transmission

**Figure 1:**
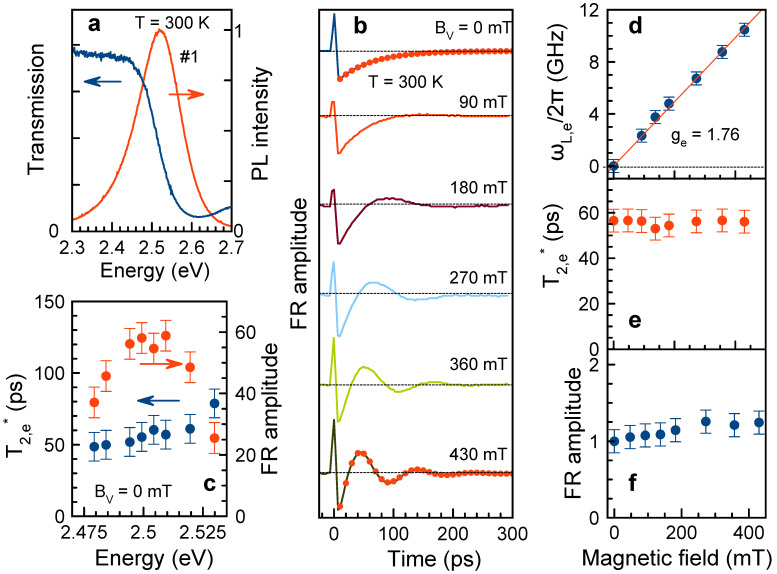
Electron spin dynamics in CsPbBr${}_{3}$ NCs (sample #1) measured at T=300 K. (**a**) Photoluminescence (red line) and transmission (blue line) spectra. (**b**) FR dynamics at different magnetic fields. Fits of the data at $B_{V}=0$ and 430 mT with Equation (2) are shown by red dots. Pump power density $P_{\mathrm{pump}}=8.4$ W/cm${}^{2}$. Laser photon energy $E_{L}=2.509$ eV. (**c**) Amplitude of FR signal (red dots) and spin relaxation time (blue dots) as a function of the laser energy near the exciton resonance. $B_{V}=0$ mT. (**d**–**f**) Magnetic field dependences of the Larmor frequency (red line shows linear fit), spin dephasing time $T_{2,e}^{*}$, and FR amplitude.

Photoluminescence and transmission spectra of sample #1 measured at a room temperature are shown in Figure 1a. The minimum in the transmission spectrum at 2.610 eV corresponds to the exciton resonance in CsPbBr${}_{3}$ NCs. The emission band is Stokes shifted from this energy, having a maximum at 2.520 eV. The PL line full width at a half maximum of 130 meV is determined by inhomogeneous broadening of the NC ensemble due to NC size dispersion.

Sample #2 has very similar PL and transmission spectra with exciton resonance slightly shifted to lower energies due to larger NC size compared to the sample #1 (see Figure 3a,b). Their evolution with the temperature in the range of 5.4–300 K is shown in the Supplementary Materials, Figure S1. We use it for evaluation of the temperature shift of the band gap energy involved in the discussion of the temperature dependence of the electron *g*-factor given below.

It is worthwhile to note, that the exciton binding energy in bulk CsPbBr${}_{3}$ amounts to 31.5 meV [39,40], which provides exciton stability in a wide temperature range. In NCs, this energy increases with decreasing the NC size [41,42].

### 3.2. Time-Resolved Faraday Rotation

Figure 1b shows the coherent spin dynamics measured by time-resolved FR in the sample #1 at room temperature. The dynamics are measured at the laser energy of $E_{L}=2.509$ eV, which corresponds to the maximum of the spectral dependence of the FR signal amplitude at a zero magnetic field, see Figure 1c. This maximum is shifted to lower energies with respect the exciton transmission minimum, as is expected for the photogeneration of the spin coherence for the resident carriers via the charged exciton (trion) resonances [26,37].

The FR dynamics (Figure 1b) are shown for transversal magnetic fields $B_{V}$ (Voigt geometry) varied from 0 up to 430 mT. The FR signals have one rapidly decaying oscillating component, which we assign to the Larmor precession of a charge carrier spins in a magnetic field. We evaluate the spin dephasing time ($T_{2}^{*}$), amplitude ($A_{0}$), and Larmor precession frequency ($\omega_{L}$) by fitting these dynamics with [37]:

(2) $$\begin{array}{c} A_{\mathrm{FR}}\left(t\right)=\sum_{e,h}A_{0,e(h)}\exp\left(-\frac{t}{T_{2,e(h)}^{*}}\right)\cos\left(\omega_{L,e(h)}t\right). \end{array}$$

Note that this equation accounts for contributions of both electrons (e) and holes (h), as both of them are typically present in the spin dynamics of bulk perovskites and their nanocrystals at cryogenic temperatures [18,26]. For fitting room temperature dynamics, we use only one contribution, and for fitting nonoscillating signal at zero magnetic field, the Larmor frequency is set to zero. Examples of the fits for magnetic fields $B_{V}=0$ and 430 mT are shown by the red dots. The evaluated parameters and their magnetic field dependences are shown in Figure 1d–f.

The dependence of the Larmor precession frequency on the magnetic field (Figure 1d) is a linear function without offset at zero field. From its slope, we evaluate the Landè factor $g_{e}=1.76\pm 0.03$ using the following expression:

(3) $$|g_{e(h)}|=\frac{\hbar \omega_{L,e(h)}}{\mu_{B}B}.$$

Here, *ℏ* is the Planck constant and $\mu_{B}$ is the Bohr magneton. Note that the electron Zeeman splitting at $B_{V}=430$ mT is 0.04 meV only, which is much smaller than the inhomogeneous broadening of the exciton transitions. Comparing this *g*-factor value with the results of Refs. [21,26,30], we assign the signal oscillations to the precession of electron spins in a transverse magnetic field. The time-resolved FR technique does not allow directly determine the *g*-factor sign. However, we firmly assign it to positive based on the papers cited above. Additional confirmation for our identification of the electron spin coherence at a room temperature comes from the observation of the hole spin precession with smaller $g_{h}=0.44$ at cryogenic temperatures, which we show below.

There are only two recent papers related to spin coherence in perovskite NCs at room temperature [27,28]. In both papers, the hole spin coherence was reported at room temperature for the CsPbBr${}_{3}$ NCs, and we observe the electron spin beats. This demonstrates that photocharging in our samples is different from the previous reports.

The evaluated spin dephasing time $T_{2}^{*}$ is about 60 ps being independent of the magnetic field strength, Figure 1e. This means that at a room temperature, it is not controlled by dispersion of *g*-factor, but rather by other spin relaxation mechanisms, among which phonon-assisted spin relaxation is most probable. The spectral dependence of the $T_{2}^{*}$ time is also very weak, Figure 1c.

The FR amplitude also weakly depends on the magnetic field, Figure 1f. We check that the amplitude increases linearly with increasing pump power, whereas electron *g*-factor and spin dephasing time are weakly sensitive to the power density in the range $P_{\mathrm{pump}}$ = 3.8–12.7 W/cm${}^{2}$ (Figure S2 in Supplementary Materials). This indicates that we perform experiments in a linear regime, despite using the laser with low repetition frequency of 25 kHz and relatively high peak power.

Similar results on electron spin coherence at room temperature are obtained for sample #2. Spin dynamics at various magnetic fields and magnetic field dependences of its parameters are shown in Figure S3 of the Supplementary Materials. The electron spin dephasing time is $T_{2,e}^{*}\approx 55$ ps and the electron *g*-factor is $g_{e}=1.69\pm 0.02$ being slightly smaller than that in the sample #1.

**Figure 2:**
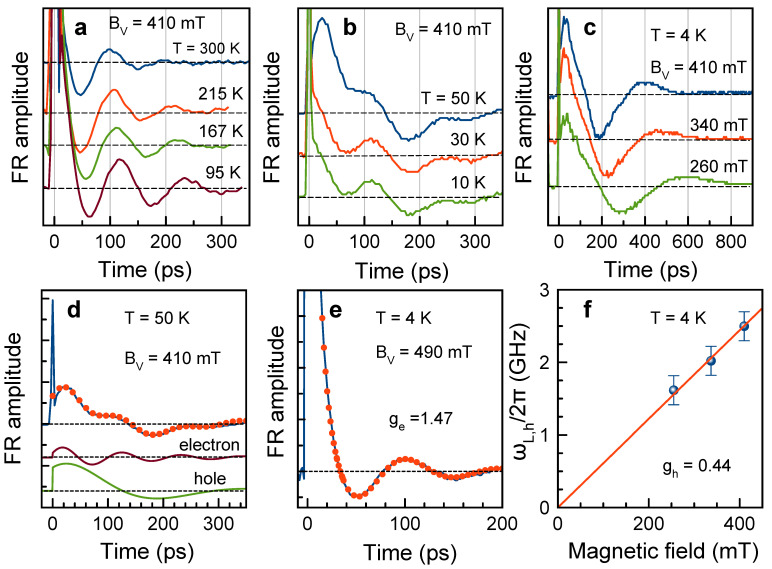
Spin dynamics of carriers in CsPbBr${}_{3}$ NCs (sample #2) measured at different temperatures. (**a**) FR dynamics in the temperature range of 95−300 K at $B_{V}=410$ mT. (**b**) FR dynamics in the temperature range of 10−50 K at $B_{V}=410$ mT. (**c**) FR dynamics at different magnetic fields at T=4 K. (**d**) FR dynamics at $B_{V}=410$ mT and T=50 K (blue line) and fit with Equation (2) (red dots). Lower panels show electron and hole contributions to the dynamics determined from the fit. (**e**) FR dynamics measured after thermal cycling: heating the sample from T=4 K, keeping it at room temperature in the darkness for several hours, and cooling in the darkness to the temperature of 4 K (blue line). Fit with Equation (2) is shown by red dots. $P_{\mathrm{pump}}=12.7$ W/cm${}^{2}$. (**f**) Magnetic field dependence of the Larmor precession frequency of holes (blue dots) and its linear fit (red line).

For sample #2, we measure the spin dynamics in a large temperature range by cooling the sample under laser illumination from 300 K down to 4 K in the magnetic field of $B_{V}=410$ mT, see Figure 2a–c. In this experiment, the laser energy is adjusted at each temperature to the maximum of the FR amplitude in order to account for the temperature shift of the exciton/trion transition.

**Figure 3:**
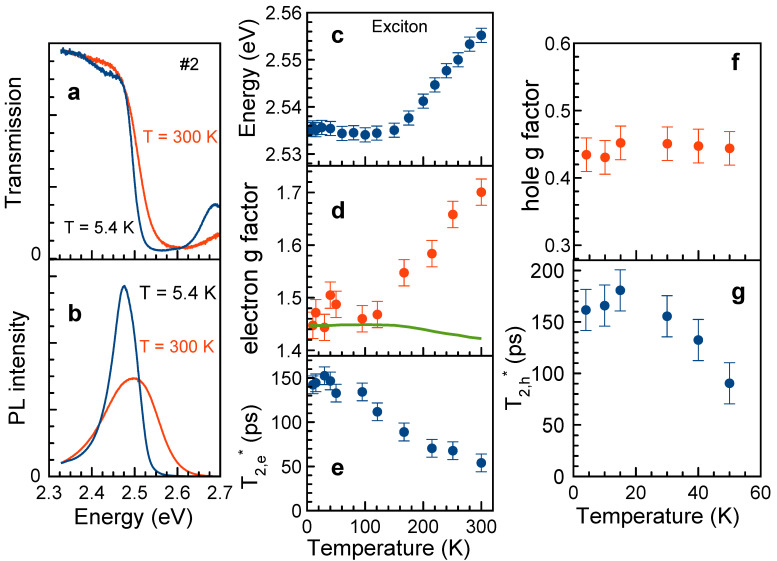
Temperature dependences of the parameters controlling spin dynamics in CsPbBr${}_{3}$ NCs (sample #2). (**a**) Transmission spectra at T=5.4 K and 300 K. (**b**) Photoluminescence spectra at T=5.4 K and 300 K. (**c**) Temperature dependence of exciton energy evaluated from the absorption spectra (see Figure S1a). (**d**) Temperature dependence of electron *g*-factor (red circles). Green line shows model expectation accounting only for the temperature shift of the band gap. (**e**) Temperature dependence of electron spin dephasing time. (**f**) Temperature dependence of hole *g*-factor. (**g**) Temperature dependence of hole spin dephasing time. Data in panels (**d**–**g**) are measured at $B_{V}=410$ mT and at $P_{\mathrm{pump}}=12.7$ W/cm${}^{2}$.

In the temperature range 95–300 K only one Larmor frequency related to the electrons is seen in the FR dynamics, Figure 2a. The Larmor precession frequency decreases with lowering temperature. At 50 K and below, a second oscillating component with lower Larmor frequency appears in the spin dynamics, Figure 2b,c. The magnetic field dependence of the lower Larmor frequency, which is evaluated by the fit with Equation (2), gives the *g*-factor $g_{h}=0.44\pm 0.01$, which we assign to holes (Figure 2f). Based on the results of Refs. [21,26,30], the hole *g*-factor is expected to be positive. The hole *g*-factor is independent of temperature in the range of 4–50 K, where the hole signal is detected, Figure 3f. The hole spin dephasing time is $T_{2,h}^{*}\approx 170$ ps at low temperatures and decreases down to 100 ps at 50 K, Figure 3g. We summarize the measured electron and hole spin dynamics parameters in Table 1. Note that in bulk CsPbBr${}_{3}$ crystals both electron and hole spin components were observed only at cryogenic temperatures with comparable, but higher *g* factors (1.96 and 0.75, respectively) [18].

Let us turn to the temperature dependences of the electron spin parameters evaluated from the FR dynamics. The electron spin dephasing time increases from 55 ps at 300 K up to 150 ps at 4 K, Figure 3e. This is expected behavior, as phonon-assisted spin relaxation mechanisms loose their efficiency at cryogenic temperatures.

Temperature dependence of the electron *g*-factor is the most interesting and unexpected finding of this study. One can see in Figure 3d, that $g_{e}=1.47$ at T=4 K and stays constant at this value for the temperature increasing up to 120 K. However, with further temperature increase, it grows nearly linearly, reaching 1.69 at 300 K. This behavior qualitatively correlates with the temperature shift of the exciton energy (Figure 3c), which in turn reflects the temperature variation of the band gap energy.

We recently experimentally demonstrated (at cryogenic temperatures) and theoretically, that the electron *g*-factor in bulk lead halide perovskites has a universal dependence on the band gap energy [21], and its value decreases with growing the band gap energy. We take the energy shift of the exciton from Figure 3c and estimate the expected changes for $g_{e}\left(T\right)$. The resulting dependence is shown by a green line in Figure 3d. One can see that the dependence is weak and small decrease in $g_{e}$ at higher temperatures is expected. This is in strong contrast with what we find experimentally. Obviously, some other mechanisms are involved here, which have an origin that is to be disclosed in future studies. It will be important to clarify experimentally whether this temperature dependence of $g_{e}$ reflects the properties of bulk perovskites or is specific for NCs. As we commented in the introduction, even for conventional semiconductors such as GaAs and CdTe, the temperature dependence of the electron *g*-factor is not fully understood. For GaAs, the possible temperature dependence of the interband matrix element was suggested [34], which in principle may be relevant for the perovskite semiconductors, but require solid experimental and/or theoretical proofs. Note that in Ref. [21] the universal dependence of the carrier *g*-factors on the band gap energy is derived assuming constant interband matrix elements. Another possible source of the $g_{e}$ temperature dependence is the involvement of the states with larger *k*-vector [33]; however, its influence should be reconsidered for NCs with strong carrier confinement. Additionally, strong electron–phonon interaction in the perovskite semiconductors may lead to temperature-dependent modification of the energy spectrum and, consequently, of the carrier *g*-factors.

We show above in Figure 2a–c, that for NCs, which are cooled rather slow and under laser illumination, the hole signal appears below 50 K as a second Larmor precession frequency. In the case when the cooling from 300 K is performed in darkness and relatively fast (during about 20 min), the hole signal does not appear, and only the electron spin precession is seen at T=4 K, Figure 2e. Obviously, photocharging of NCs either by electrons or by holes is responsible for this phenomenon [43]. Note that time-resolved FR is a very suitable technique for identifying the type of the resident carriers in colloidal NCs by measuring the Larmor precession of the specific carrier. We have demonstrated this for CdSe- and CdS-based colloidal NCs [44,45,46]. For CdS NCs, we have shown that the photocharging can develop dynamically from negative to positive [44]. The photocharging physics is complex and is determined by many factors (concentration and parameters of surface states, efficiency of Auger processes for electrons and holes, matrix in which NCs are embedded, lattice temperature, cooling and illumination conditions, etc.). Our results show that depending on illumination conditions during the cooling process we can realize either negatively photocharged NCs or coexistence of the negatively and positively charged NCs. In both cases, the presence of neutral (empty) NCs is also expected; however, these do not appear in the time-resolved FR experiments.

### 3.3. Differential Transmission and Optical Orientation Dynamics

To obtain further insight in the population and spin dynamics, we use the time-resolved differential transmission technique. The measurements were performed for sample #1 at room temperature and at zero magnetic field. The laser energy of $E_{L}=2.509$ eV is the same as in experiments with the time-resolved FR presented in Figure 1.

**Figure 4:**
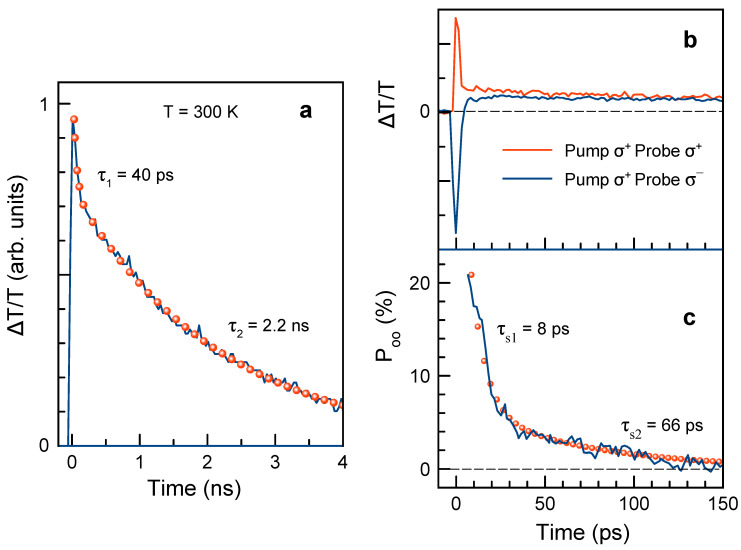
Time-resolved differential transmission measurements of CsPbBr${}_{3}$ NCs (sample #1) at T=300 K. (**a**) Population dynamics measured with linearly polarized pump ($P_{\mathrm{pump}}=5.1$ W/cm${}^{2}$) and linearly polarized probe. Red dots show two-exponential fit. $E_{L}=2.509$ eV. (**b**) Spin dynamics measured for $\sigma^{+}$ polarized pump and for either $\sigma^{+}$ (red line) or $\sigma^{-}$ (blue line) polarized probe. $P_{\mathrm{pump}}=12.7$ W/cm${}^{2}$. (**c**) Dynamics of optical spin orientation degree (blue line) calculated for data from panel (**b**). Dots show two-exponential fit.

The population dynamics, measured with linearly polarized pump and probe beams, are shown in Figure 4a. Initially, about 20% of the signal amplitude decays with time $\tau_{1}=40$ ps and further signal decays with $\tau_{2}=2.2$ ns. We study the ensemble of CsPbBr${}_{3}$ NCs, where neutral NCs coexist with NCs charged by either electrons or holes. The neutral NCs gives rise to the dynamics of (neutral) excitons, and the charged NCs to the trion dynamics. Recombination dynamics of excitons in lead halide perovskite NCs at room temperature falls in the range of 1–2 ns [47,48,49]. Therefore, we assign the longer time of 2.2 ns in our NCs to the lifetime of excitons in neutral NCs. The shorter time of 40 ps can be assigned to trion lifetime in charged NCs, which is shortened by efficient Auger process. From the ratio of the amplitudes of the exciton and trion decays in Figure 4a we evaluate that about 20% of the NCs are charged and 80% stay neutral at room temperature.

Spin dynamics can be also addressed by the time-resolved differential transmission technique when the circularly polarized pump and probe are used. The circularly polarized pump, for which we take $\sigma^{+}$ polarization, photogenerates spin-polarized excitons/trions or charge carriers. Dynamics of their spin polarization are probed by transmission of either $\sigma^{+}$ or $\sigma^{-}$ circularly polarized probe. The respective dynamics are shown in Figure 4b. These dynamics are similar to those measured in Refs. [19,50], except for a negative signal that we observe for a very short time. It is presumably related to the non-linear effects taking place for time-overlapping pump and probe pulses. The underlying mechanisms of carrier spin orientation are the same as in the time-resolved FR experiments and described in Ref. [37]. From these dynamics, we calculate the optical orientation degree using Equation (1), and these dynamics are displayed in Figure 4c. The $P_{\mathrm{oo}}\left(t\right)$ dynamics have two spin relaxation times of $\tau_{s1}=8$ ps and $\tau_{s2}=66$ ps. The longer time coincides with $T_{2,e}^{*}=60$ ps time that we measure by the time-resolved FR, Figure 1e. Based on that, we assign it to the spin relaxation of resident electrons in negatively charged NCs. The faster time of 8 ps can be either related to the spin relaxation of excitons in neutral NCs or to the hole spin relaxation in negatively charged trions. We suggest that the exciton spin relaxation is more probable here.

We are aware of only one report where the spin dynamics in perovskite (CsPbI${}_{3}$) NCs have been measured by the time-resolved differential transmission [19]. Measured spin relaxation times of 3 ps at room temperature and 32 ps at cryogenic temperatures were attributed to charge carriers. In this experiment, the carriers were photogenerated with a large excess energy, as the laser photon energy was considerably detuned from the exciton resonance. This is an important difference from the conditions of our experiment, where the resonant excitation was used. In our experiment, we measured 66 ps at a room temperature for the spin relaxation dynamics of the resident electrons at the bottom of the conduction band.

Application of the time-resolved differential transmission technique to the bulk lead halide perovskites, such as MAPbI${}_{3}$, CsPbI${}_{3}$, MAPbBr${}_{3}$, and CsPbBr${}_{3}$ polycrystalline films, evidence very fast spin dynamics of <5 ps [11,20]. Note that the data for bulk structures should be taken with care when comparison with NCs is made, as zero-dimensional confinement strongly modifies the available spin relaxation mechanisms of carriers and excitons.

## 4. Conclusions

We have studied carrier spin dynamics in CsPbBr${}_{3}$ perovskite nanocrystals by means of the time-resolved Faraday rotation and differential transmission techniques. Coherent spin dynamics in form of the Larmor spin precession of electrons and holes are provided by the NCs singly photocharged by these carriers. The photocharging process depends on whether the sample is cooled under laser illumination or in darkness. The coherent spin precession of electrons is observed in the temperature range from 4 K up to room temperature. Their spin dephasing time of 60 ps at room temperature is rather long compared to that for conventional semiconductors. Unexpected temperature dependence of the electron *g*-factor, which increases with growing temperature and with increasing the band gap energy, is found. The coherent spin dynamics of holes emerge at cryogenic temperatures of 4–50 K. Our results demonstrate that lead halide perovskite NCs are very interesting and promising materials for spintronics and information technologies exploiting spin-dependent phenomena.

## Figures and Tables

**Table 1:** Spin parameters measured for CsPbBr${}_{3}$ perovskite NCs at different temperatures. $T_{2}^{*}$ values are given for $B_{V}=410$ mT.

	300 K	4 K
Electron *g*-factor, $g_{e}$	1.77	1.47
Hole *g*-factor, $g_{h}$	–	0.44
Electron spin dephasing time, $T_{2,e}^{*}$	60 ps	150 ps
Hole spin dephasing time, $T_{2,h}^{*}$	–	170 ps

## Data Availability

The data presented in this study are available on request from the corresponding author.

## Supplementary Materials

The following supporting information can be downloaded at: https://www.mdpi.com/article/10.3390/nano13172454/s1, Figure S1: photoluminescence and transmission spectra of the sample #2 measured in a temperature range 5.4–300 K; Figure S2: dependences of spin parameters on the pump power density in the sample #1; Figure S3: experimental data for time-resolved FR studies of the sample #2.
