# Supplementary material for: Coherent Spin Dynamics of Electrons in CsPbBr3 Perovskite Nanocrystals at Room Temperature
Source: Nanomaterials (Basel). 2023 Aug 30;13(17):2454. doi: 10.3390/nano13172454 (PMC10489742; doi:10.3390/nano13172454)
Supplement: Supplementary file 1 [file nanomaterials-13-02454-s001.zip › nanomaterials-2575802-supplementary.pdf]

Supplementary Materials: Coherent spin dynamics of electrons in $CsPbBr_3$ perovskite nanocrystals at room temperature

Sergey R. Meliakov, Evgeny A. Zhukov, Evgeniya V. Kulebyakina, Vasilii V. Belykh, Dmitri R. Yakovlev

S1. PHOTOLUMINESCENCE AND ABSORPTION AT DIFFERENT TEMPERATURES

In order to measure the temperature dependence of the band gap, we measure the temperature shift of the exciton transition in transmission/absorption spectra. The absorption spectra for the temperature range from 5.4 K up to 300 K are shown in Figure S1a. One can see that the spectra ares contributed by several lines. We separate them by using fit with three Gaussian peaks, which are shown by dashed lines. We assign the lowest in energy peak (green dashed one) to the exciton resonance and take its maximum for plotting the temperature shift in Figure 3c of the main text.

For completeness, we measure and show in Figure S1b the PL spectra in the same temperature range. For measuring the transmission spectra the intensity of the transmitted light ($I_{\rm tr}$) was normalized to the intensity of the incident light (I_0). In Figure S1a the absorption spectra at different temperatures were calculated by the following expression:

$$\alpha d = ln(I_{\rm tr}/I_0), \quad \text{(S1)}$$

here α is absorption coefficient, and d is thickness of the absorbing layer. At low temperatures, the absorption spectra have a complex shape provided by exciton, trion and exciton excited states. Therefore, the fit of the absorption spectra is performed by three Gaussians.

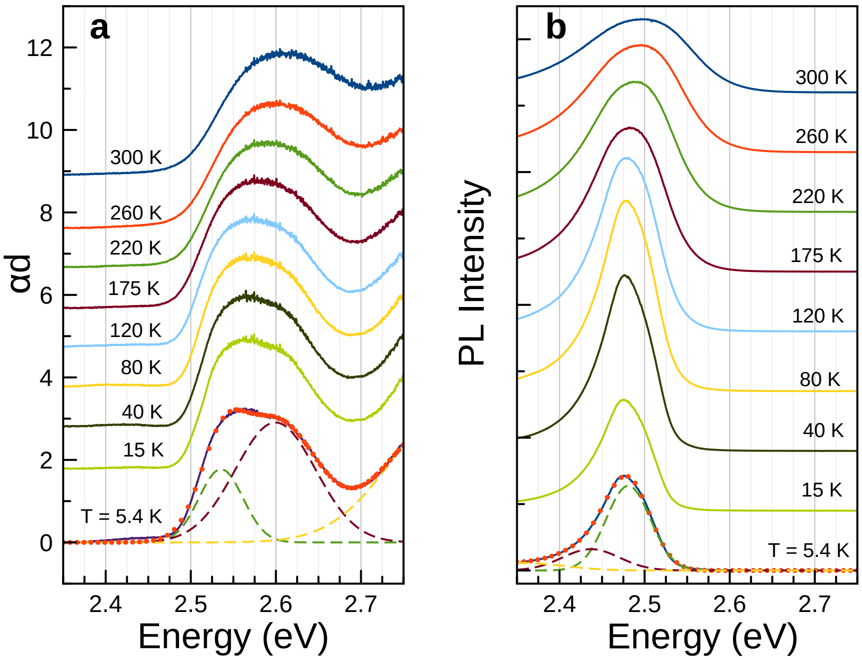

Figure S1. Absorption **(a)** and photoluminescence **(b)** spectra at different temperatures for the sample #2. Red dots show fits to absorption and photoluminescence spectra at $T = 5.4$ K using three Gaussian peaks (dashed lines of different colors). The absorption is plotted in units of $\alpha d = \ln(I_{\rm tr}/I_0)$, where I_0 and $I_{\rm tr}$ are incident and transmitted intensities, respectively, d is the sample thickness and α is the absorption coefficient.

S2. DEPENDENCE OF SPIN DYNAMICS PARAMETERS ON PUMP POWER DENSITY

We analyse the spin dynamics measured for pump power densities in the range of $P_{\mathrm{pump}} = 3.8 - 12.7$ W/cm^2. The respective spin parameters (FR amplitude, electron g-factor and electron spin dephasing time) are shown in Figure S2. The FR amplitude increases almost linearly with the pump power (Figure S2a), while the frequency of the Larmor precession (electron g factor) and the dephasing time are unchanged within experimental errors (Figures S2b,c). These results prove that we are in the linear regime on the excitation power density for the performed experiments.

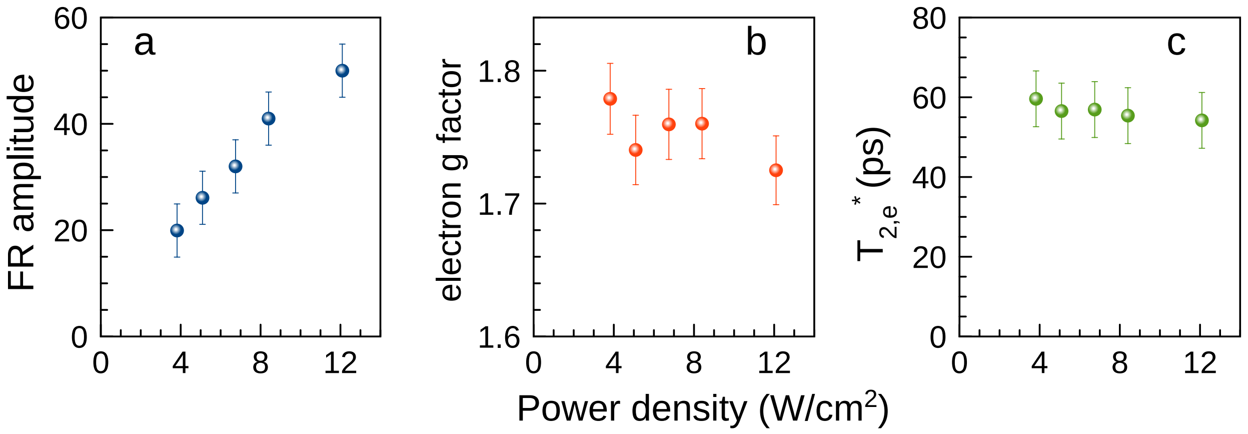

Figure S2. Pump power density dependences of FR signal amplitude **(a)**, electron g factor **(b)**, and electron spin dephasing time **(c)** measured for the sample #1. $T = 300$ K, $B_{\mathrm{V}} = 270$ mT, $E_{\mathrm{L}} = 2.509$ eV, and $P_{\mathrm{probe}} = 8.9$ W/cm^2.

S3. SPIN DYNAMICS IN SAMPLE #2

Here we show results on spin dynamics for the sample #2, similar to the results for the sample #1 displayed in the main text in Figure 1.

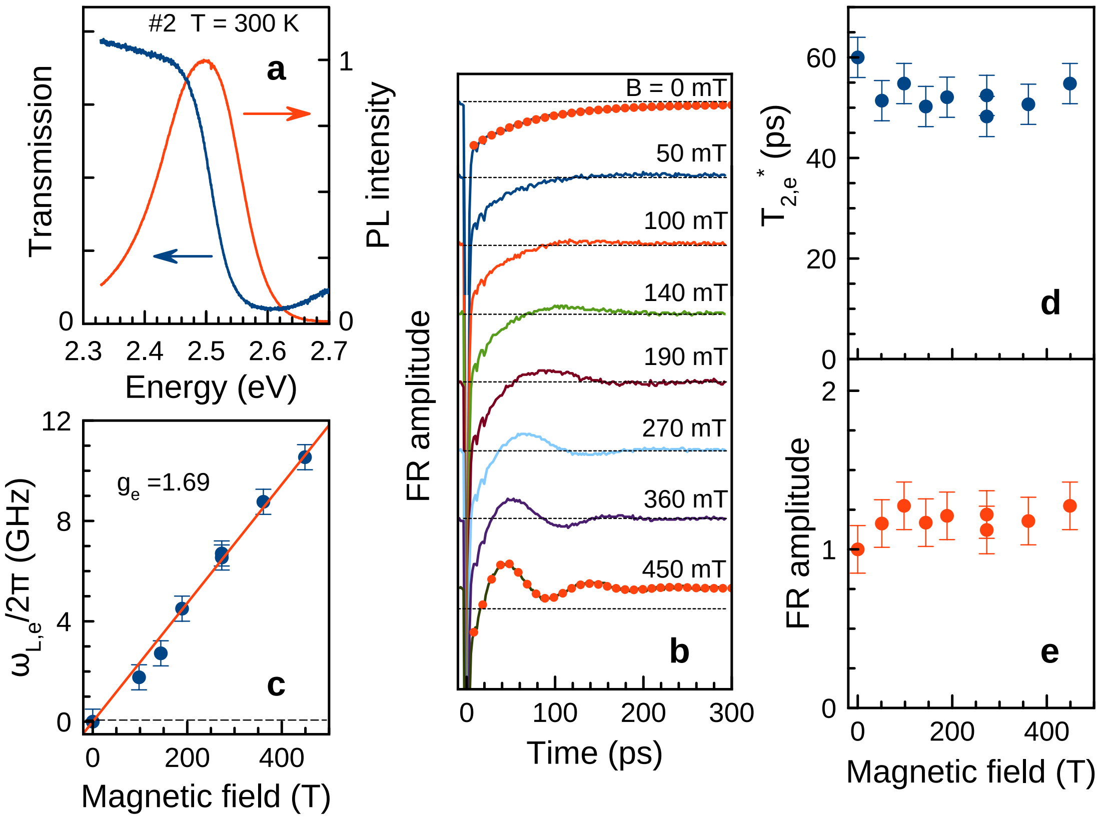

Figure S3. Spin dynamics of carriers in $CsPbBr_3$ NCs (sample #2) measured at $T = 300$ K. **(a)** Photoluminescence (red line) and absorption (blue line) spectra. **(b)** Time-resolved FR signal at different magnetic fields. Fit of the data at $B_V = 0$ and 450 mT with Eq. (2) (red dots). $P_{pump} = 7.6$ W/cm^2. $E_L = 2.499$ eV. **(c),(d),(e)**Magnetic field dependences of the Larmor frequency [linear fit of these data (red line)], spin dephasing time $T^*_{2,e}$, and FR amplitude.
